# Validation of an algorithm that determines stroke diagnostic code accuracy in a Japanese hospital-based cancer registry using electronic medical records

**DOI:** 10.1186/s12911-017-0554-x

**Published:** 2017-12-04

**Authors:** Yasufumi Gon, Daijiro Kabata, Keichi Yamamoto, Ayumi Shintani, Kenichi Todo, Hideki Mochizuki, Manabu Sakaguchi

**Affiliations:** 1 Department of Neurology, Osaka University Graduate School of Medicine, 2-2, Yamadaoka, Suita, Osaka, 565-0871 Japan; 2 Department of Medical Statistics, Osaka City University Graduate School of Medicine, Osaka, Japan; 3 Department of Drug and Food Clinical Evaluation, Osaka City University Graduate School of Medicine, Osaka, Japan

**Keywords:** Electronic medical record, Diagnostic code, Validation, Clinical research

## Abstract

**Background:**

This study aimed to validate an algorithm that determines stroke diagnostic code accuracy, in a hospital-based cancer registry, using electronic medical records (EMRs) in Japan.

**Methods:**

The subjects were 27,932 patients enrolled in the hospital-based cancer registry of Osaka University Hospital, between January 1, 2007 and December 31, 2015. The ICD-10 (international classification of diseases, 10th revision) diagnostic codes for stroke were extracted from the EMR database. Specifically, subarachnoid hemorrhage (I60); intracerebral hemorrhage (I61); cerebral infarction (I63); and other transient cerebral ischemic attacks and related syndromes and transient cerebral ischemic attack (unspecified) (G458 and G459), respectively. Diagnostic codes, both “definite” and “suspected,” and brain imaging information were extracted from the database. We set the algorithm with the combination of the diagnostic code and/or the brain imaging information, and manually reviewed the presence or absence of the acute cerebrovascular disease with medical charts.

**Results:**

A total of 2654 diagnostic codes, 1991 “definite” and 663 “suspected,” were identified. After excluding duplicates, the numbers of “definite” and “suspected” diagnostic codes were 912 and 228, respectively. The proportion of the presence of the disease in the “definite” diagnostic code was 22%; this raised 51% with the combination of the diagnostic code and the use of brain imaging information. When adding the interval of when brain imaging was performed (within 30 days and within 1 day) to the diagnostic code, the proportion increased to 84% and 90%, respectively. In the algorithm of “definite” diagnostic code, history of stroke was the most common in the diagnostic code, but in the algorithm of “definite” diagnostic code and the use of brain imaging within 1 day, stroke mimics was the most frequent.

**Conclusions:**

Combining the diagnostic code and clinical examination improved the proportion of the presence of disease in the diagnostic code and achieved appropriate accuracy for research. Clinical research using EMRs require outcome validation prior to conducting a study.

## Background

The introduction of electronic databases in the medical field is rapidly progressing. Accordingly, a large amount of electronic data has become available for the study of clinical medicine, health services, and population health [1–3]. Electronic databases have already been utilized in clinical epidemiology research, such as linking with large-scale health care databases in Europe and the United States [4–8].

Electronic medical records (EMRs) in Japan were introduced in 2002 as part of the national strategy for the conversion of medical information technology. The implementation rate of the EMRs in all hospitals throughout Japan (*n* = 8605) in 2011 was approximately 20%. Implementation was especially evident in hospitals with 400 beds or more (*n* = 822 hospitals), with rates exceeding 50% [9]. However, the history of EMRs in Japan is relatively short, and they are often underutilized in clinical research. EMRs contain an array of valuable patient-care information for the purpose of supporting research and quality health care [10]. The re-use of EMR data for clinical research provides large datasets with long-term observations at low cost, enabling researchers to conduct studies effectively and efficiently [2, 11, 12]. Conversely, it has also been reported that research using diagnostic codes, such as the ICD-10 (international classification of diseases, 10th revision) [13] of EMRs has inherent problems. Specifically, it is unknown whether a patient actually has a registered disease, even if there is a registered diagnostic code [14–16]. Namely, EMRs have problems in the large variation of coding practices between clinicians [14]. If diagnostic codes are analyzed as outcomes in isolation, certain diseases may be overestimated and lead to erroneous results [17, 18]. Therefore, validation of outcomes prior to conducting a study is essential for clinical research using EMRs [11].

Previous studies have suggested that patients with cancer are at high risk of stroke [19–21], and stroke in patients with cancer are reported to have unique clinical characteristics [22–24]. However, limited data is available on the epidemiological aspects between cancer and stroke. As many types of cancer exist, conducting exhaustive research between cancer and stroke is challenging. The number of cancer survivors continuously increases with the progress of cancer care, and therefore, research that focuses on the relationships between cancer and other diseases are required for the future care. In this regard, the current study posited that the use of EMRs, which contain an enormous amount of patient information with long follow-up times, could aid in the investigation of epidemiological aspects between cancer and stroke. The purpose of this study was to validate an algorithm that determines the accuracy of stroke diagnostic codes, in a hospital-based cancer registry using EMRs, prior to conducting epidemiological research between cancer and stroke in Japan.

## Methods

### Subjects and study setting

Subjects were patients enrolled in the hospital-based cancer registry of Osaka University Hospital, between January 1, 2007 and December 31, 2015. Osaka University Hospital is a large, academic, urban hospital that functions as a regional comprehensive cancer center, which typically accepts more patients with cancer than a local hospital. In Japan, the Cancer Control Act was enacted in 2006 [25], and other nation-based cancer registry projects have also been launched. Cancer registration is subdivided into three types of registries: hospital-based cancer registry, population-based cancer registry, and organ-based cancer registry. A hospital-based cancer registry is a registry that collects clinical data, including cancer type and stage, on all patients treated for cancer in a comprehensive cancer hospital, along with a population-based cancer registry. All patients receive regular follow-ups until death, and patients who are referred for a second opinion are excluded from the registry. A total of 27,932 patients with cancer were identified during the study period. Of these, 2105 patients were excluded because 2083 were duplicates and 22 displayed an input mistake (i.e., date of death was before date of birth). Therefore, 25,827 patients with cancer (all Asian; 49.5% women; age 61.6 ± 15.7 years) were included in the study cohort. Using this cancer cohort and EMR database, we investigated the presence of the diagnostic codes for stroke and validated an algorithm that examines the accuracy of diagnostic codes, with the combination of diagnostic codes and/or clinical information.

### Extraction of medical information from EMRs

The ICD-10 diagnostic codes for stroke were extracted from the EMR database. Specifically, subarachnoid hemorrhage (I60); intracerebral hemorrhage (I61); cerebral infarction (I63); and other transient cerebral ischemic attacks and related syndromes and transient cerebral ischemic attack (unspecified) (G458 and G459), respectively. The EMR database in Japan has two types of diagnostic designations: “definite,” which is the code where the final diagnosis is definite, and “suspected,” which is the code where the final diagnosis is suspected. In the current study, both of these codes were obtained. First, stroke diagnostic codes entered in the hospital-based cancer registry were examined. Then, in order to assess the improvement of the accuracy of diagnostic codes by adding clinical information, use of brain imaging (computed tomography and/or magnetic resonance imaging) were also obtained from the EMR database. Additionally, factors related to stroke were obtained from the EMR as follows: hypertension (I10–I15); diabetes mellitus (E10–E14); dyslipidemia (E78); atrial fibrillation (I48); and brain metastases (C793).

### Algorithm to determine the accuracy of diagnostic codes

**Fig. 1:**
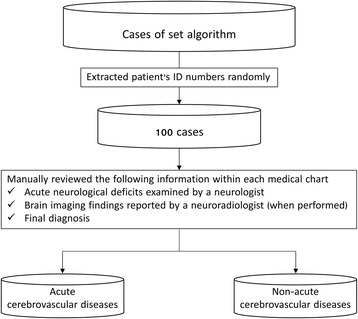
Flow of reviewing medical charts

In clinical practice, there is the possibility that more than one different department may register the same diagnostic codes. Therefore, we first located and removed duplicate registrations, in order to avoid an overestimation of the presence of the disease. Duplicate registration of a diagnostic code was defined as when two or more of the same diagnostic codes are registered on the same day. Next, we set the algorithm, with the combination of diagnostic codes and/or clinical information as follows: 1) “definite” diagnostic code; 2) “definite” diagnostic codes and the use of brain imaging; 3) “definite” diagnostic code and the use of brain imaging performed within 30 days of diagnosis; 4) “definite” diagnostic code and the use of brain imaging performed within 1 day of diagnosis; and 5) “suspected” diagnostic code and the use of brain imaging. In each algorithm, 100 cases were extracted using uniform random numbers generated by computer, and manually reviewed for the presence of the registered disease within the medical charts. We also examined the position of the diagnostic code, primary, secondary, or others. We defined the presence of acute cerebrovascular disease in advance by having a clinically specific description of disease. Specially, we collected the following information in each medical chart: the description of acute neurological deficit examined by a neurologist; brain imaging findings reported by a neuroradiologist (when performed); and final diagnosis. Based on these pieces of information, we identified the presence or absence of disease (Fig. 1). All procedures were performed with R software (https://www.r-project.org) using the “rms” package [26].

This study was approved by the ethics committee for clinical research at Osaka University Hospital, Osaka, Japan. The need for informed consent was waived, due to the retrospective nature of the study.

## Results

Table 1 shows the baseline characteristics of the study cohort. Among 25,827 patients who were enrolled in the hospital-based cancer registry, breast cancer was the most common form of cancer, followed by uterine, colorectal, gastric, and lung cancer. The number of patients with stage 0, I, II, III, and IV was 1512 (6.1%), 7680 (31.2%), 3873 (15.8), 2830 (11.5%), and 3809 (15.5%), respectively. Four hundred and seventy-three patients (1.8%) had brain metastases. The number of stroke risk factors including hypertension, diabetes mellitus, dyslipidemia, and atrial fibrillation was 5230 (20.3%), 4532 (17.5%), 3287 (12.7%), and 861 (3.3%), respectively.

**Table 1:** Baseline Characteristics

Characteristics	*N* = 25,827
Age, year	61.5 ± 15.7
Male, % (*n*)	50.4 (13025)
Type of cancer, % (*n*)	
Breast	11.3 (2906)
Uterus	9.1 (2366)
Colorectal	8.1 (2097)
Gastric	7.9 (2030)
Lung	6.5 (1683)
Brain	6.3 (1634)
Prostate	6.3 (1626)
Esophageal	5.4 (1383)
Hepatic	4.3 (1118)
Oropharyngeal	4.0 (1033)
Others^a^	
Stage of cancer^b^, % (*n*)	
0	6.1 (1512)
I	31.2 (7686)
II	15.8 (3873)
III	11.5 (2830)
IV	15.5 (3809)
Unknown	20.9 (5144)
Brain metastases, % (*n*)	1.8 (471)
Stroke risk factors, % (*n*)	
Hypertension	20.3 (5230)
Diabetes mellitus	17.5 (4532)
Dyslipidemia	12.7 (3287)
Atrial fibrillation	3.3 (861)

^a^Include hematopoietic malignancies (*n* = 973), pancreatic cancer (*n* = 806), skin cancer (*n* = 795), renal cancer (*n* = 653), thyroid cancer (*n* = 652), ovarian cancer (*n* = 494), bladder cancer (*n* = 475), malignant lymphoma (*n* = 439), biliary tract cancer (*n* = 327), laryngeal cancer (*n* = 303), soft connective tissue cancer (*n* = 277), spinal cord tumors (*n* = 256), cancer of unknown primary origin (*n* = 145), eye cancer (*n* = 137), peritoneal cancer (*n* = 128), bone cancer (*n* = 125), small intestinal cancer (*n* = 124), maxillary sinus cancer (*n* = 106), testicular cancer (*n* = 94), mediastinal cancer (*n* = 88), ureter cancer (*n* = 83), nasal cancer (*n* = 80), vulvar cancer (*n* = 63), parathyroid cancer (*n* = 36), vaginal cancer (*n* = 36), penile cancer (*n* = 24), scrotal cancer (*n* = 23), pineal tumor (*n* = 21), anus cancer (*n* = 19), tracheal cancer (*n* = 9), peripheral nerve tumor (*n* = 7), and choriocarcinoma (*n* = 4)

^b^Breakdown of 24,854 cases with solid cancer (excluding hematopoietic malignancies)

**Fig. 2:**
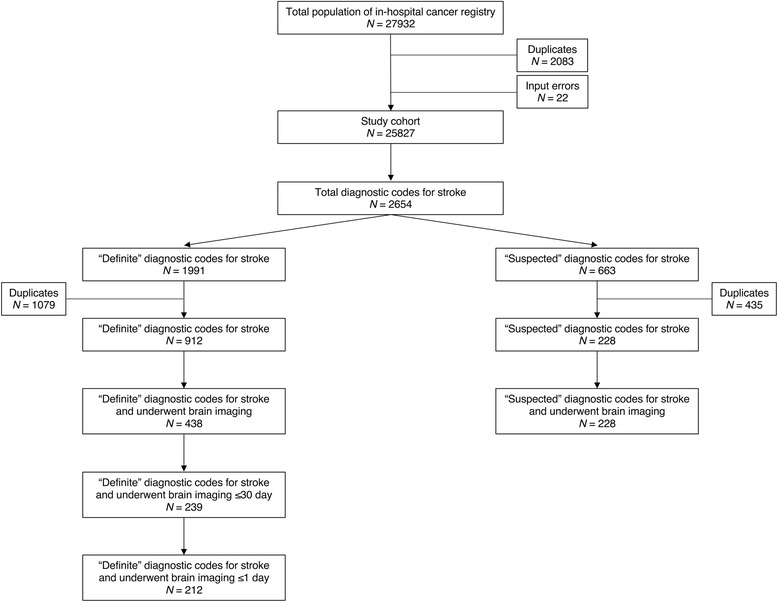
Working flow of subjects in the validation study. A total of 27,932 patients with cancer were identified during the study period. Of these, 2105 patients were excluded because 2083 were duplicate registration and 22 had an input mistake (die before being born). Therefore, 25,827 patients with cancer were included in the study cohort

A total of 2654 diagnostic codes were identified from the EMR database. Of these, the number of “definite” and “suspected” diagnostic codes were 1991 and 663, respectively. The number of brain imaging orders was 4544. After removing duplicates, the numbers of “definite” and “suspected” diagnostic codes were 912 and 228, respectively. The number of each algorithm of “definite” diagnostic code, “definite” diagnostic code and the use of brain imaging, “definite” diagnostic code and the use of brain imaging performed within 30 days of diagnosis, and “definite” diagnostic code and the use of brain imaging within 1 day of diagnosis were 912, 438, 239, and 212 records, respectively. The number of “suspected” diagnostic code and the use of brain imaging was 228 records. Details are shown in Fig. 2.

Table 2 shows the validation results of diagnostic code accuracy. In the algorithm of the “definite” diagnostic code, the proportion of the presence of the disease was 22%. The proportion of the presence of the disease in the algorithm of “definite” and the use of brain imaging was 51%. When adding the interval of when brain imaging was performed within 30 days and 1 day of diagnosis to the “definite” diagnostic code, the proportion of the presence of the disease was 84% and 90%, respectively. In the algorithm of the “suspected” diagnostic code and the use of brain imaging, the proportion of the disease was 7%. In the algorithm of the “definite” diagnostic code, the primary diagnostic position was more accurate than the secondary or other diagnostic position (57% vs 19%). In the algorithm of the “definite” diagnostic code and the use of brain imaging within 30 days and 1 day, both positions represented high accuracy (84% vs 90%).

**Table 2:** Validation Results of Diagnostic Codes Accuracy

Algorithm	Number of diagnostic codes, *n*	Number of acute cerebrovascular diseases, *n*	Proportion, %
“Suspected” diagnostic code and the use of brain imaging			
All	100	7	7
“Definite” diagnostic code			
All	100	22	22
Primary	7	4	57
Secondary or others	93	18	19
“Definite” diagnostic code and the use of brain imaging			
All	100	51	51
Primary	7	4	57
Secondary or others	93	47	51
“Definite” diagnostic code and the use of brain imaging within 30 days			
All	100	84	84
Primary	8	6	75
Secondary or others	92	78	85
“Definite” diagnostic code and the use of brain imaging within 1 day			
All	100	90	90
Primary	7	6	86
Secondary or others	93	84	90

Table 3 shows the breakdown of non-acute cerebrovascular diseases. In the algorithm of “definite” diagnostic code, history of stroke was the most common in the diagnostic code, but in the algorithm of “definite” diagnostic code and the use of brain imaging within 1 day, stroke mimics was the most frequent.

**Table 3:** Breakdown of Non-acute Cerebrovascular Diseases

	“Definite” diagnostic code (*n* = 78)	“Definite” diagnostic code and the use of brain imaging (*n* = 49)	“Definite” diagnostic code and the use of brain imaging within 30 days (*n* = 16)	“Definite” diagnostic code and the use of brain imaging within 1 day (*n* = 10)
History of stroke, *n*	41	16	3	1
Stroke mimics, *n*	22	17	10	9
Cervical spondylotic myelopathy	4	2	1	–
Seizure	4	1	–	–
Dementia	4	–	–	–
Disturbance of consciousness	2	2	3	3
Syncope	2	1	2	2
Vestibular dysfunction	2	1	1	–
Brain tumor	1	6	1	1
Acute mononeuropathy	1	1	–	–
Toxic/Metabolic symptoms	1	–	–	1
Functional or medically unexplained symptoms	1	–	–	–
Sudden severe headache	–	2	1	1
Ophthalmic disorder	–	1	1	1
Blood and/or imaging tests, *n*	15	16	3	–

## Discussion

We validated an algorithm to determine the accuracy of stroke diagnostic codes, in a hospital-based cancer registry in Japan, using EMRs. The proportion of the presence of acute cerebrovascular diseases in the diagnostic code for stroke was approximately 20%. This improved when disease-specific, clinical examination information was added to the diagnostic code; approximately half of the diagnostic codes exhibited the disease with the combination of the diagnostic code and the use of brain imaging. Further, this number was raised to over 80% when the brain imaging performed within 30 days of the diagnosis.

EMRs provide valuable patient-care information for the purpose of research and health care information [11]. However, EMRs are primarily built for clinical care process, and have inherent issues of data reliability for use in medical research. That is, EMRs have problems in the large variation in coding practices between clinicians [17]. More recently, Williams et al. suggested recommendations for research using electronic medical data [12]. One of the essential steps mentioned is the validation of setting outcomes to reduce potential type I errors, where an incorrect code is wrongly included [12]. We accordingly validated the setting outcomes, and identified that the proportion of the presence of the disease in definite diagnostic code was approximately 20% in our study cohort. This means that the outcome would be overestimated approximately five times more than the actual number if only the diagnostic code was directly measured. Previous studies have reported differences of up to seven times higher, depending on the outcome setting [17, 18], and our results support this. In clinical research using electronic databases, incorrect outcome settings can lead to erroneous results. This study confirmed the importance of outcome validation in research using EMRs, and suggested the usefulness of combining the clinical examination to the diagnostic code, rather than using the diagnostic code in isolation.

In this study, the proportion of the presence of disease using only the EMR diagnostic code was approximately 20%. This percentage was lower than expected. There are several potential reasons why only one in five of the diagnostic codes actually demonstrated the disease. First, there are problems of large variation in coding practices between clinicians. For example, some clinicians may register diagnostic codes only for blood and/or imaging tests. Unfortunately, this behavior causes type I error. In addition, as previously mentioned, this disparity may be due to not inputting data to EMRs from the viewpoint of research. The second reason concerns when the diagnostic code was registered. A previous study has reported that stroke classification in administrative data were optimal using all discharge diagnoses for ischemic stroke and primary discharge diagnosis for intracerebral and subarachnoid hemorrhage [27]. In this study, we extracted the diagnostic code from the EMR, irrespective of when it was registered. Manual review revealed that, in most cases, the diagnostic code was registered at symptom onset or on admission. Therefore, it was speculated that the differences between the studies were when the diagnostic code was registered, discharge or symptom onset. Finally, the history of EMRs in Japan is relatively recent, and clinicians may be unfamiliar with the registration of the diagnostic code. Indeed, the overall introduction rate of EMR in Japan was only 20% in 2011. Further, as this study was conducted in a single facility, it may not reflect the current state of affairs throughout Japan. However, it is suggested that the presence of the diagnostic code does not necessarily mean the presence of the registered disease in EMRs. It seems necessary for clinicians to improve awareness of registration of diagnostic codes.

A previous study has suggested that the accuracy of diagnostic codes differ depending on the position of diagnostic code [27]. In this study, the difference between primary, and secondary or other diagnostic positions were evident in the algorithm of “definite” diagnostic code. This difference was the narrowest in the algorithm of “definite” diagnostic code and the use of brain imaging within 30 and 1 day. The reason the proportion of the primary diagnostic position was lower than that of secondary or other diagnostic positions in the “definite” diagnostic code and use of brain imaging within 30 days is unclear. However, the variation in coding practices between clinicians may account for these results.

A strength of the current study is that it actually identified the description of the disease in each patient chart and validated the algorithms. However, there are also several limitations to this study. First, performing such a manual review is a time-consuming process. Therefore, it is unlikely that all applied studies could utilize this approach [9]. Thus, a more efficient method of outcome validation is needed in future. Second, we did not examine the presence of the disease in all cases, but rather in randomly extracted cases with the diagnostic codes. Thus, we could not perform sensitivity analysis between the algorithm and the presence of the disease. Third, our data was limited to Japanese EMRs from within a single center. Thus, our findings may not generalize to other EMRs. Fourth, the EMR contains information on patients treated at Osaka University hospital and does not cover patients admitted to other hospitals when they had developed stroke. Additionally, stroke is a clinical diagnosis in many cases, and stroke that has not been coded cannot be collected by using EMRs. These characteristics lead a possibility of underestimating the incidence of stroke. Finally, the proportion of the presence of the diseases in the diagnostic codes may also change if the disease and facilities are different. Therefore, there is a need to combine different disease-specific clinical assessments and different diagnostic codes.

## Conclusions

Combining the diagnostic code and the clinical examination improved the proportion of the presence of the disease in the diagnostic code and achieved sufficiently high accuracy to conduct research. However, outcomes will likely be overestimated if EMR diagnostic codes are utilized in isolation. Therefore, clinical research using EMRs should validate outcomes prior to conducting a study.

## References

[1] Casey JA, Schwartz BS, Stewart WF, Adler NE (2016). Using electronic health records for population health research: a review of methods and applications. Annu Rev Public Heatlh.

[2] Goldstein BA, Navar AM, Pencina MJ, Ioannidis JP (2017). Opportunities and challenges in developing risk prediction models with electronic health records data: a systematic review. J Am Med Inform Assoc.

[3] Prokosch HU, Ganslandt T (2009). Perspectives for medical informatics. Reusing the electronic medical record for clinical research. Methods Inf Med.

[4] Brownstein JS, Murphy SN, Goldfine AB, Grant RW, Sordo M, Gainer V (2010). Rapid identification of myocardial infarction risk associated with diabetes medications using electronic medical records. Diabetes Care.

[5] Denny JC, Ritchie MD, Crawford DC, Schildcrout JS, Ramirez AH, Pulley JM (2010). Identification of genomic predictors of atrioventricular conduction: using electronic medical records as a tool for genome science. Circulation.

[6] Chen DP, Morgan AA, Butte AJ (2010). Validating pathophysiological models of aging using clinical electronic medical records. J Biomed Inform.

[7] Kullo IJ, Fan J, Pathak J, Savova GK, Ali Z, Chute CG (2010). Leveraging informatics for genetic studies: use of the electronic medical record to enable a genome-wide association study of peripheral arterial disease. J Am Med Inform Assoc.

[8] Elkhenini HF, Davis KJ, Stein ND, New JP, Delderfield MR, Gibson M (2015). Using an electronic medical record (EMR) to conduct clnical trials: Salford lung study feasibility. BMC Med Inform Decis Mak.

[9] Ministry of Internal Affairs and Communications (2015). White paper informations and communications in Japan. http://www.soumu.go.jp/johotsusintokei/whitepaper/ja/h27/html/nc121320.html. [Japanese] Accessed 29 May 2017.

[10] Blumenthal D, Tavenner M (2010). The “meaningful use” regulation for electronic health records. N Engl J Med.

[11] Wiliams R, Kontopantelis E, Buchan I, Peek N (2017). Clinical code set engineering for reusing EHR data for research: a review. J Biomed Inform.

[12] Yamamoto K, Sumi E, Yamazaki T, Asai K, Yamori M, Teramukai S (2012). A pragmatic method for electronic medical record-based observational studies: developing an electronic medical records retrieval system for clinical research. BMJ Open.

[13] World Health Organization (WHO). International statistical classification of diseases and related health problems, tenth revision. Geneva. World Health Organization. 1994. 3376487

[14] Ancker JS, Kern LM, Edwards A, Nosal S, Stein DM, Hauser D (2014). How is the electronic health record being used? Use of EHR data to assess physician-level variability in technology use. J Am Med Inform Assoc.

[15] Calvert M, Shankar A, McManus R, Lester H, Freemantle N (2009). Effect of the quality and outcomes framework on diabetes care in the United Kingdom: retrospective cohort study. BMJ.

[16] Hogan WR, Wagner MM (1997). Accuracy of data in computer-based patients records. J Am Med Inform Assoc.

[17] Rodríguez LA, Tolosa LB, Ruigómez A, Johansson S, Wallander MA (2009). Rheumatoidarthritis in UK primary care: incidence and prior morbidity. Scand J Rheumatol.

[18] Watson DJ, Rhodes T, Guess HA (2003). All-cause mortality and vascular events among patients with rheumatoid arthritis, osteoarthritis, or no arthritis in the UK general practice research database. J Rheumatol.

[19] Navi BB, Reiner AS, Kamel H, Iadecola C, Elkind MS, Panageas KS (2015). Association between incident cancer and subsequent stroke. Ann Neurol.

[20] Zöller B, Ji J, Sundquist J, Sundquist K (2012). Risk of haemorrhagic and ischaemic stroke in patients with cancer: a nationwide follow-up study from Sweden. Eur J Cancer.

[21] Chen PC, Muo CH, Lee YT, YH Y, Sung FC (2011). Lung cancer and incidence of stroke: a population-based cohort study. Stroke.

[22] Gon Y, Sakaguchi M, Takasugi J, Kawano T, Kanki H, Watanabe A (2017). Plasma D-dimer levels and ischaemic lesions in multiple vascular regions can predict occult cancer in patients with cryptogenic stroke. Eur J Neurol.

[23] Gon Y, Okazaki S, Terasaki Y, Sasaki S, Yoshimine T, Sakaguchi M (2016). Characteristics of cryptogenic stroke in cancer patients. Ann Clin Transl Neurol.

[24] Schwarzbach CJ, Schaefer A, Ebert A, Held V, Bolognese M, Kablau M (2012). Stroke and cancer: the importance of cancer-associated hypercoagulation as a possible stroke etiology. Stroke.

[25] Overview of the "Cancer Control Act". http://www.mhlw.go.jp/english/wp/wp-hw3/dl/2-077.pdf. Accessed 18 June 2017.

[26] Package 'rms'. https://cran.r-project.org/web/packages/rms/rms.pdf. Accessed 27 May 2016.

[27] Tirschwell DL, Longstreth WT Jr. Validating administrative data in stroke research. Stroke 2002;33:2465–2470. 10.1161/01.str.0000032240.28636.bd 12364739

